# A multi-national cross-sectional exploration of rehabilitation services for children and young people following brain injury in low and middle income countries

**DOI:** 10.1186/s12889-025-24933-0

**Published:** 2025-11-03

**Authors:** Mark A. Linden, Leigh Schrieff-Brown, Linda Ewing-Cobbs, R. A. Leonard, Rajendra Prasad, Juan Carlos Arango-Lasprilla, Sandra Strazzer, Lucia Braga, Consuelo Reyes Payeras, Kim C. Davis, Lisa Kalungwana-Mambwe, Flavia Dorrego, Mathilde Chevignard

**Affiliations:** 1 School of Nursing and Midwifery, Queen’s University Belfast, 97 Lisburn Road, Belfast, BT9 7BL UK; 2 Department of Psychology, University of Cape Town, Cape Town, South Africa; 3 Department of Pediatrics and Children’s Learning Institute McGovern Medical School, University of Texas Health Science Center at Houston, Houston, USA; 4 Department of Neurosurgery, Indraprastha Apollo Hospitals, New Delhi, India; 5 Department of Cell Biology and Histology, University of the Basque Country, Bilbao, Spain; 6 Acquired Brain Injury Unit, Scientific Institute IRCCS E. Medea, Bosisio Parini, Italy; 7 Neuropsychology, Neurosciences, and Neurorehabilitation Department, SARAH Network of Rehabilitation Hospitals, Brasilia, Brazil; 8 Centro de Estudios en Neurociencia Humana y Neuropsicología, Facultad de Psicología, Universidad Diego Portales, Santiago, Chile; 9 Department of Pediatrics, Baylor College of Medicine, Houston, USA; 10 Department of Psychology, University of Zambia, Lusaka, Zambia; 11 Child Neuropsychology, Fundación de Lucha contra Enfermedades Neurológicas de la Infancia, Buenos Aires, Argentina; 12 Rehabilitation Department for children with acquired neurological injury, Saint Maurice Hospitals, Saint Maurice, Paris, France; 13 Laboratoire d’Imagerie Biomédicale, Sorbonne Université, LIB, Inserm, CNRS, Paris, 75006 France; 14 Groupe de Recherche Clinique n°24 Handicap Cognitif et Moteur et Réadaptation (HaMCRe), Sorbonne Université, Paris, France

**Keywords:** Survey, Cross-sectional, Low and middle income countries, Service provision, Rehabilitation, Children and young people, Brain injury, Health

## Abstract

**Background:**

Brain injury (BI) is the largest cause of mortality and morbidity among children and can lead to significant cognitive, social, emotional and behavioural deficits. There has been an absence of research examining the availability of rehabilitation services for affected children and young people in low and middle income countries (LMICs). This study therefore investigated current rehabilitation provision in LMICs for children and young people with BI.

**Methods:**

An online survey was developed which collected data on funding support for rehabilitation, causes of BI and access to services. The survey was distributed to healthcare professionals known to members of the research team. Participants were asked to forward the survey to other professionals in their networks. Representation was sought from as many LMICs as possible. Data were analysed using descriptive statistics (e.g. percentages) and the Kruskal Wallis test to explore differences between Official Development Assistance (ODA) status (least developed, lower middle and upper middle) on access to services.

**Results:**

A total of 179 participants from 32 LMICs responded to the survey. Healthcare professionals from the least developed countries reported charities as the main source of funding to support rehabilitation, while those from upper middle income regions were most often funded by governments. According to healthcare professionals, the greatest cause of BI in LMICs was hypoxia. The Kruskal Wallis test comparing ODA status showed statistically significant differences in access to services for occupational (*P* = .005), speech and language (*P* = .038) and aquatic therapies (*P* < .001), cognitive (*p* < .001) and vocational rehabilitation (*P* = .008), and community based inclusive development programmes (*P* = .009).

**Conclusions:**

There is a lack of equity in accessibility of rehabilitation services for children and young people with BI in LMICs, with some services being non-existent in lower income countries. Improvements to obstetric services, screening and management of infectious diseases are needed to reduce rates of hypoxic injuries and injury prevention programmes are needed to reduce traumatic injury. Centralised, government funding is required to develop services to improve life chances for these children.

**Supplementary Information:**

The online version contains supplementary material available at 10.1186/s12889-025-24933-0.

## Introduction

 Brain injury (BI) is a lifelong disability which can result in cognitive, behavioural, social, emotional and physical deficits [1–4]. The overarching category of BI includes congenital and birth-related injuries as well as BI acquired following birth as a consequence of infection, disease, stroke, hypoxia and trauma [5]. The multifaceted nature of BI and its varied aetiologies complicate efforts to establish precise prevalence rates, particularly in paediatric populations. A recent World Health Organization report examined the global burden of disorders affecting the nervous system. Across ages 0–19, neonatal encephalopathy, neurological complications due to preterm birth, encephalitis, meningitis, epilepsy, stroke, and nervous system cancer were among the top 10 conditions accounting for the greatest disability-adjusted life years [6]. Within the acquired BI spectrum, traumatic brain injury (TBI) stands out as a critical global health concern, with an estimated 69 million cases annually. Low-resource regions bear a disproportionate share of this burden, amplifying the need for targeted interventions in these settings [7].

Early and effective rehabilitation has been consistently shown to improve outcomes for children and young people with brain injury [7–10]. Early intervention is critical to optimizing recovery and reducing long-term disability. However, access to timely and adequate rehabilitation services remains a significant challenge, particularly in low-resource settings. In such regions, systemic barriers—including a lack of specialised care and trained healthcare workforces [6]—exacerbate the already devastating consequences of BI, leading to poor functional outcomes and a diminished quality of life for affected individuals [10–12].

To contextualize these disparities, it is essential to consider the global landscape of healthcare access and funding. The Development Assistance Committee (DAC) of the Organization for Economic Cooperation and Development (OECD) lists 140 countries which are eligible for Official Development Assistance (ODA), based on gross national income (GNI) per capita as published by the World Bank [11]. These countries, collectively referred to as low- and middle-income countries (LMICs), are classified into four subcategories: least developed, low-income, middle-income, and upper-middle-income countries [13]. The OECD, comprising 37 developed economies, plays a pivotal role in fostering sustainable economic growth and addressing inequalities in resource allocation, including healthcare.

Despite the substantial global burden of paediatric BI, there has been an absence of research examining the availability of rehabilitation services for affected children and young people in LMICs. To address this gap, this study sought to examine the accessibility of rehabilitation provision in LMIC for children and young people with BI. Data were also collected on how rehabilitation was funded in different regions of the world. As countries may have different rehabilitation needs and different BI aetiologies the profile of BI causes across countries was also explored.

## Methods

### Design

This was a cross-sectional study, utilising an online survey via the platform, Microsoft Forms. The survey was translated from English into Spanish, Portuguese and French.

### Participants and setting

Representation was sought from as many countries as possible from the DAC list of ODA countries (*n* = 140). Only two countries qualify for the ODA ranking of low income, the Democratic People’s Republic of Korea and the Syrian Arab Republic. It was not possible to recruit participants from these countries meaning that three out of the four ODA categories were represented in this study (e.g. least developed, middle-income, and upper-middle-income countries).

Both convenience and snowball sampling utilising existing networks of professionals from LMICs. Healthcare professionals working in the area of BI were targeted, as these were the most likely participants to be involved with current rehabilitation provision for children and young people. Participation was open to individuals across all levels of professional experience and roles. No restrictions were placed on years in practice, area of expertise (e.g. medical specialists, allied health or community professionals), or time spent in-country for those trained internationally. To be eligible, participants had to be from a country from one of the ODA categories and be familiar with rehabilitation services in their country. All participants voluntarily self-selected to complete the survey.

### Measure

**Fig. 1:**
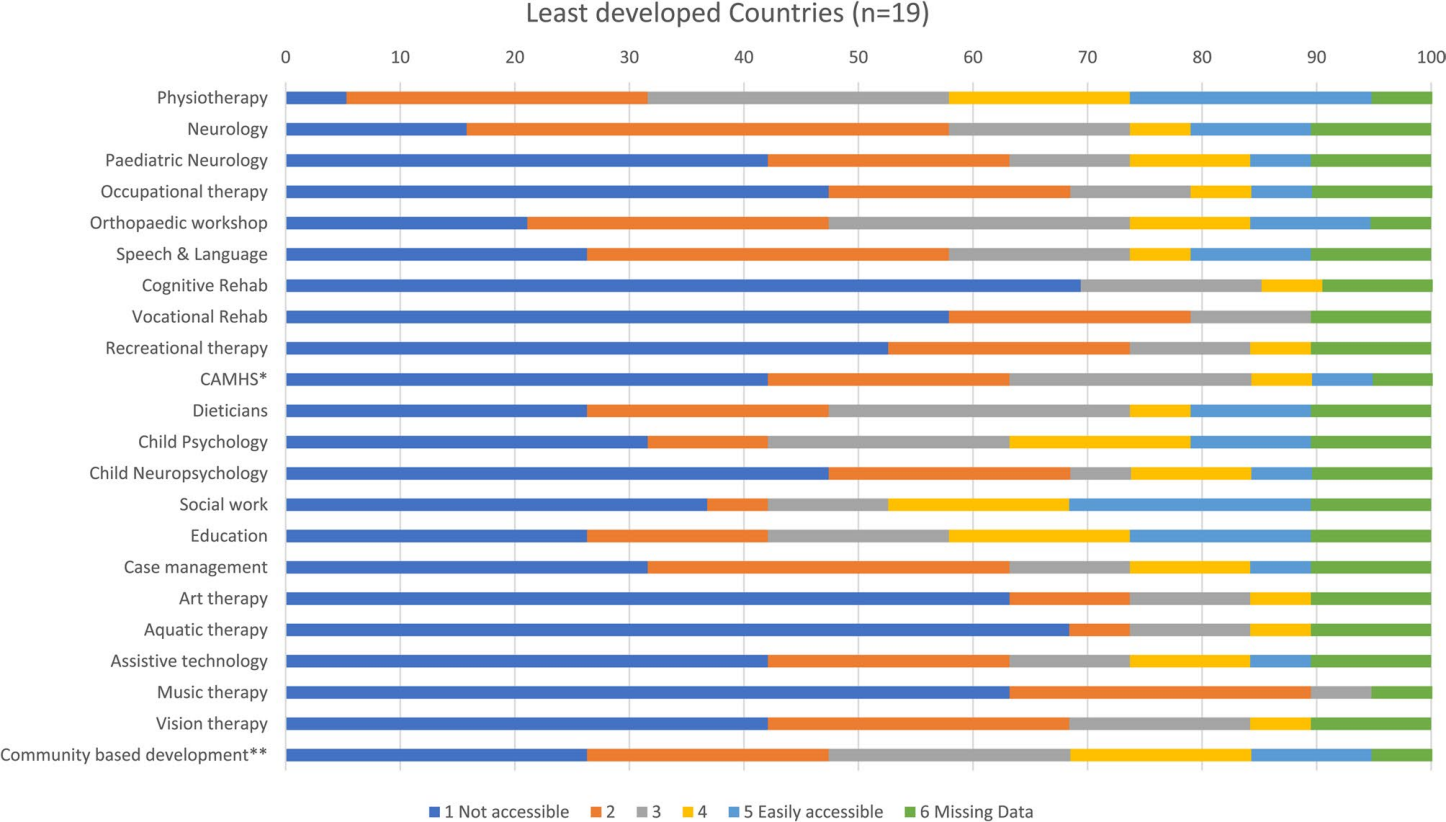
Stacked bar chart showing responses for access to services in least developed countries. *child and adolescent mental health services; **community based inclusive development programme

As there is no existing measure of access to services for paediatric BI in LMIC, the research team created their own survey for this study. The survey was developed based on the expert opinion of twelve international rehabilitation professionals from 11 countries. Seven individuals were themselves working in LMICs. Colleagues utilised an online platform to meet over a period of 3 months to develop and refine survey content. The final version of the survey was agreed upon by all authors before proceeding to pilot testing. The survey was then piloted with three healthcare professionals prior to distribution to assess phrasing, understanding and readability. These professionals comprised two neuropsychologists (USA and South Africa) and a neurosurgeon (India) with between 13 and 39 years of experience in the field. Alterations were made to wording by the international team of authors to improve clarity and ensure similar terms were employed across all regions (see supplementary file for the survey). For example, colleagues working in LMIC were familiar with community based inclusive development programmes which were unknown to those from developed countries. These programmes were subsequently added to the list of rehabilitation services. Participants were asked to score how easy it was for children and young people to access a range of services on a five-point Likert scale from 1 = not accessible to 5 = easily accessible. Services included physiotherapy, occupational therapy, neurology, clinical psychology, dieticians, education, cognitive rehabilitation etc. For a complete list of services, see Fig. 1. Further, participants were asked to report on who paid for services, what the main causes of BI were and what barriers to rehabilitation existed in their countries. Data were also collected on sex, qualifications, profession, years of rehabilitation experience and location of hospital/rehabilitation centre where the participant worked (rural versus urban).

### Ethical approval

Ethical approval was granted by the Faculty of Medicine Health and Life Sciences research ethics committee at the Queen’s University Belfast in Northern Ireland (Ref, MHLS 23_117). Participants were informed that all data provided would be held in confidence and that it would not be possible to identify them in any subsequent publication. All participants provided informed consent prior to taking part in the survey.

### Data collection

Data were collected between September to November 2023. The survey was created in English and then translated into French, Spanish and Portuguese using the Microsoft Word 365 translation tool. These translations were then checked, and modifications made, by native French, Spanish and Portuguese speakers. These versions were back translated into English to check on accuracy of the language. An email invitation and participant information sheet, containing a link to the survey, were sent to potential participants working in LMICs. Participants read the information sheet and indicated their consent to participate by checking a box in relation to a number of consent questions, prior to completing the survey.

### Analysis

Descriptive statistics, employing stacked bar charts were used to display percentage of access to services for least developed, lower middle, upper middle and non-ODA countries. Causes of BI, causes of TBI and sources of rehabilitation funding are displayed as percentages via bar charts. The independent samples Kruskal Wallis test was used to test for differences between ODA status (least developed, lower middle and upper middle) on access to services. Pairwise comparisons between statistically significant findings were used to further explore where these differences arose. As non-ODA countries comprised only 5 responses, this group was omitted from statistical analysis. An alpha level of 0.05 was established a priori to control for Type I error. All analysis was conducted using IBM SPSS for statistics version 22.0 [12].

## Results

Responses were received from 184 individuals from 34 countries, however, 5 were ineligible as they came from non ODA territories (Vietnam *n*=2 and Chile *n*=3). The sample therefore comprised 32 LMIC or 22.8% of all LMICs on the ODA list. See Table 1 for respondent characteristics. Participants comprised 140 females and 39 males, with the majority of responses coming from upper middle (*n*=142), followed by least developed (*n*=19) and lower middle income (*n*=18) countries. A large proportion of participants came from Brazil (*n*=64). The largest group of respondents were medics/physicians (*n*=46), followed by physiotherapists (*n*=34), nurses (*n*=22), neuropsychologists (*n*=17) and psychologists (*n*=15). The majority of participants worked in urban settings (*n* = 170), with 14 working in rural settings. Further, 61% of participants had more than 5 years of rehabilitation experience, with 55% working in paediatric settings for more than 5 years.

**Table 1:** Characteristics of respondents by country and ODA ranking

	Country	Gender	Profession	Rural/Urban	Highest qualification
Least developed (*n*=19)	Bangladesh (*n*=2)	Female (*n*=2)	Nursing (*n*=2)	Rural (*n*=1), Urban (*n*=1)	Postgraduate (*n*=1), Doctorate (*n*=1)
	Bhutan (*n*=1)	Female (*n*=1)	Physiotherapy (*n*=1)	Urban (*n*=1)	Postgraduate (*n*=1)
	Ethiopia (*n*=2)	Male (*n*=2)	Nursing (*n*=1), Social work (*n*=1)	Urban (*n*=2)	Postgraduate (*n*=2)
	South Sudan (*n*=3)	Female (*n*=2), Male (*n*=1)	Physiotherapy (*n*=3)	Rural (*n*=1), Urban (*n*=2)	Baccalaureate (*n*=2), Postgraduate (*n*=1)
	Sudan (*n*=6)	Female (*n*=6)	Physiotherapy (*n*=5), Rehabilitation assistant (*n*=1)	Urban (*n*=6)	Certificate (*n*=1), Baccalaureate (*n*=5)
	Yemen (*n*=2)	Male (*n*=2)	Physiotherapy (*n*=1), Rehabilitation assistant (*n*=1)	Rural (*n*=2)	Diploma (*n*=1), Postgraduate (*n*=1)
	Zambia (*n*=3)	Female (*n*=1), Male (*n*=2)	Education (*n*=3)	Urban (*n*=3)	Baccalaureate (*n*=2), Postgraduate (*n*=1)
Lower middle (*n*=18)	Bolivia (*n*=3)	Female (*n*=2), Male (*n*=1)	Physiatry (*n*=1), Psychology (*n*=2)	Urban (*n*=3)	Baccalaureate (*n*=1), Postgraduate (*n*=2)
	Egypt (*n*=1)	Female (*n*=1)	Education (*n*=1)	Rural (*n*=1)	Doctorate (*n*=1)
	Ghana (*n*=5)	Female (*n*=4), Male (*n*=1)	Education (*n*=1), Midwifery (*n*=4)	Rural (*n*=1), Urban (*n*=4)	Diploma (*n*=1), Baccalaureate (*n*=2), Postgraduate (*n*=1), Doctorate (*n*=1)
	Honduras (*n*=1)	Female (*n*=1)	Neuropsychology (*n*=1)	Urban (*n*=1)	Postgraduate (*n*=1)
	India (*n*=6)	Female (*n*=4), Male (*n*=2)	Occupational therapy (*n*=1), Physiotherapy (*n*=5)	Urban (*n*=6)	Baccalaureate (*n*=2), Postgraduate (*n*=4)
	Mongolia (*n*=1)	Female (*n*=1)	Nursing (*n*=1)	Urban (*n*=1)	Postgraduate (*n*=1)
	Nicaragua (*n*=1)	Male (*n*=1)	Physiatry (*n*=1)	Urban (*n*-1)	Postgraduate (*n*=1)
Upper middle (*n*=142)	Argentina (*n*=5)	Female (*n*=5)	Neuropsychology (*n*=1)	Urban (*n*=5)	Diploma (*n*=1), Postgraduate (*n*=3), Doctorate (*n*=1)
	Armenia (*n*=1)	Male (*n*=1)	Medic (*n*=1)	Urban (*n*=1)	Doctorate (*n*=1)
	Botswana (*n*=1)	Female (*n*=1)	Neuropsychology (*n*=1)	Urban (*n*=1)	Doctorate (*n*=1)
	Brazil (*n*=64)	Female (*n*=59), Male (*n*=5)	Education (*n*=8), Medic (*n*=14), Neuropsychology (*n*=1), Nursing (*n*=16), Occupational therapy (*n*=2) Physiotherapy (*n*=11), Psychology (*n*=8), Speech and language therapy (*n*=4)	Urban (n=64)	Baccalaureate (n=22), Postgraduate (n=37), Doctorate (n=5)
	China (*n*=5)	Female (*n*=3), Male (*n*=2)	Nursing (*n*=1), Medic (*n*=4)	Rural (*n*=1), Urban (*n*=4)	Postgraduate (*n*=5)
	Colombia (*n*=9)	Female (*n*=5), Male (*n*=4)	Neuropsychology (*n*=7), Physiatry (*n*=1), Rehabilitation assistant (*n*=1)	Rural (*n*=2), Urban (*n*=7)	Postgraduate (*n*=8), Doctorate (*n*=1)
	Dominican Republic (*n*=1)	Male (*n*=1)	Physiatry (*n*=1)	Urban (*n*=1)	Postgraduate (*n*=1)
	Ecuador (*n*=2)	Female (*n*=2)	Physiatry (*n*=1), Physiotherapy (*n*=1)	Urban (*n*=2)	Diploma (*n*=1), Postgraduate (*n*=1)
	Georgia (*n*=1)	Female (*n*=1)	Medic (*n*=1)	Urban (*n*=1)	Postgraduate (*n*=1)
	Guatemala (*n*=9)	Female (*n*=4), Male (*n*=5)	Education (n=1) Medic (n=1), Neuropsychology (*n*=3), Physiatry (*n*=1), Psychology (*n*=3)	Rural (*n*=1), Urban (*n*=8)	Baccalaureate (*n*=1), Postgraduate (*n*=8)
	Jordan (*n*=3)	Female (*n*=2), Male (*n*=1)	Physiotherapy (*n*=2), Nursing (*n*=1)	Rural (*n*=1), Urban (*n*=2)	Baccalaureate (*n*=2), Doctorate (*n*=1)
	Libya (*n*=1)	Female (*n*=1)	Physiotherapy (*n*=1)	Rural (*n*=1)	Diploma (*n*=1)
	Mexico (*n*=29)	Female (*n*=24), Male (*n*=5)	Medic (*n*=22), Neuropsychology (*n*=1), Occupational therapy (*n*=1), Physiatry (*n*=3), Physiotherapy (*n*=1), Psychology (*n*=1)	Rural (*n*=1), Urban (*n*=28)	Baccalaureate (*n*=1), Postgraduate (*n*=26), Doctorate (*n*=2)
	Moldova (*n*=1)	Female (*n*=1)	Medic (*n*=1)	Rural (*n*=1)	Postgraduate (*n*=1)
	Panama (*n*=1)	Female (*n*=1)	Neuropsychology (*n*=1)	Urban (*n*=1)	Doctorate (*n*=1)
	Peru (*n*=3)	Female (*n*=3)	Medic (*n*=2), Psychology (*n*=1)	Urban (*n*=3)	Postgraduate (*n*=3)
	South Africa (*n*=3)	Female (*n*=2), Male *(n=1)*	Neuropsychology *(n=1),* Educator (*n*=2)	Urban (*n*=3)	Postgraduate (*n*=1), Doctorate (*n*=2)
	Turkey (*n*=3)	Female (*n*=1), Male (*n*=2)	Physiotherapy (*n*=3)	Urban (*n*=3)	Baccalaureate (*n*=2), Postgraduate (*n*=1)
Non-ODA responses (*n*=5)	Chile (*n*=3)	Female (*n*=3)	Occupational therapy (*n*=1), Psychology (*n*=2)	Urban (*n*=3)	Postgraduate (*n*=3)
	Vietnam (*n*=2)	Female (*n*=2)	Medic (*n*=2)	Urban (*n*=2)	Postgraduate (*n*=1), Doctorate (*n*=1)

### Causes of ABI by ODA ranked region this should be presented first before the above comparison or relation between groups

**Fig. 2:**
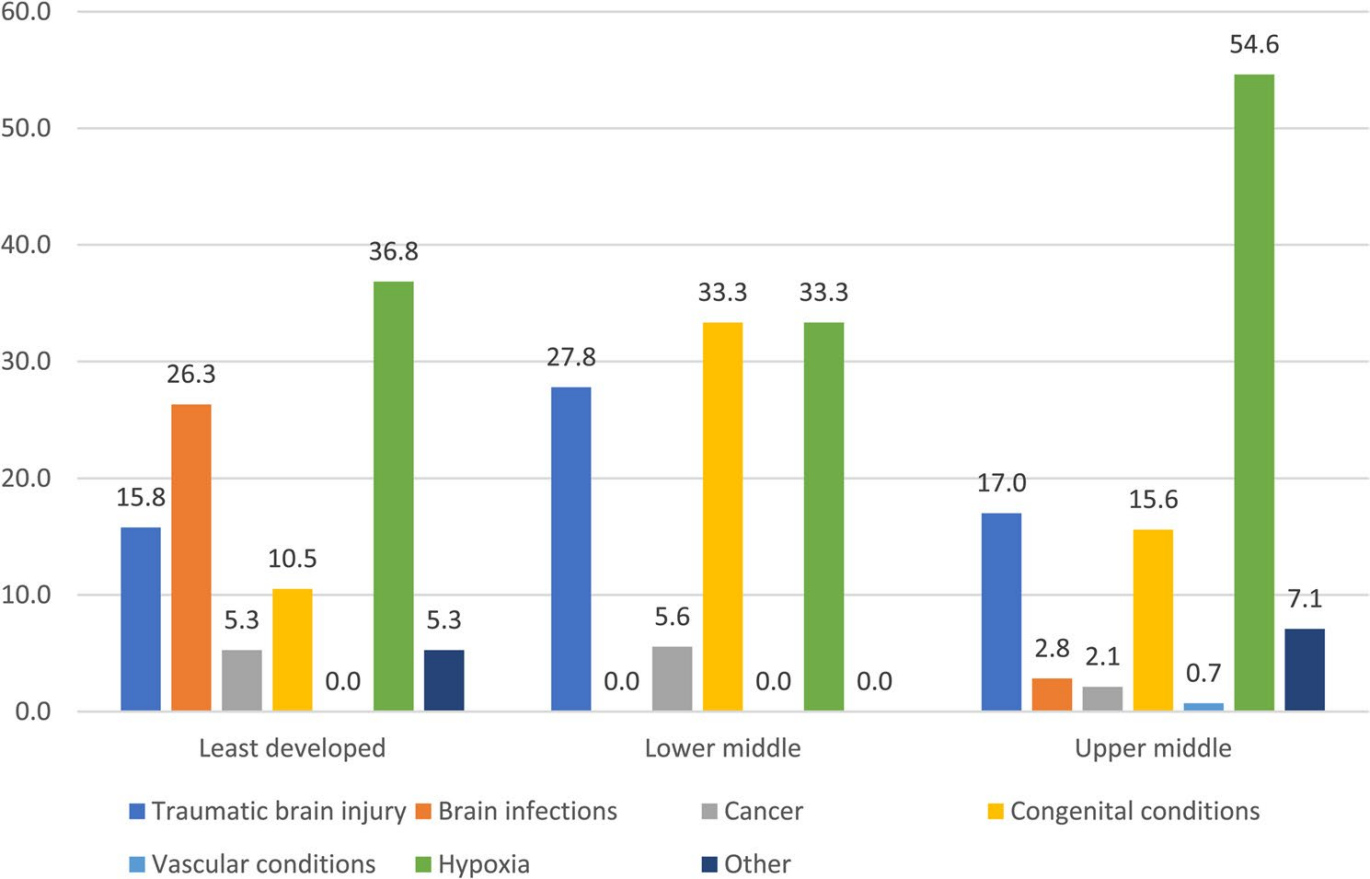
Cause of ABI as percentages by ODA regions

Healthcare professionals from least developed countries (see Fig. 2) reported that the majority of BIs arose from hypoxic injuries (37%) followed by brain infections (26%) and trauma (16%). Colleagues from lower middle income countries reported that congenital conditions (33%) and hypoxic injuries (33%) were the most common followed by trauma (28%). The main cause of BI in upper middle income countries was hypoxia (55%) followed by trauma (17%) and congenital conditions (16%).

### Cause of TBI by ODA ranked region

**Fig. 3:**
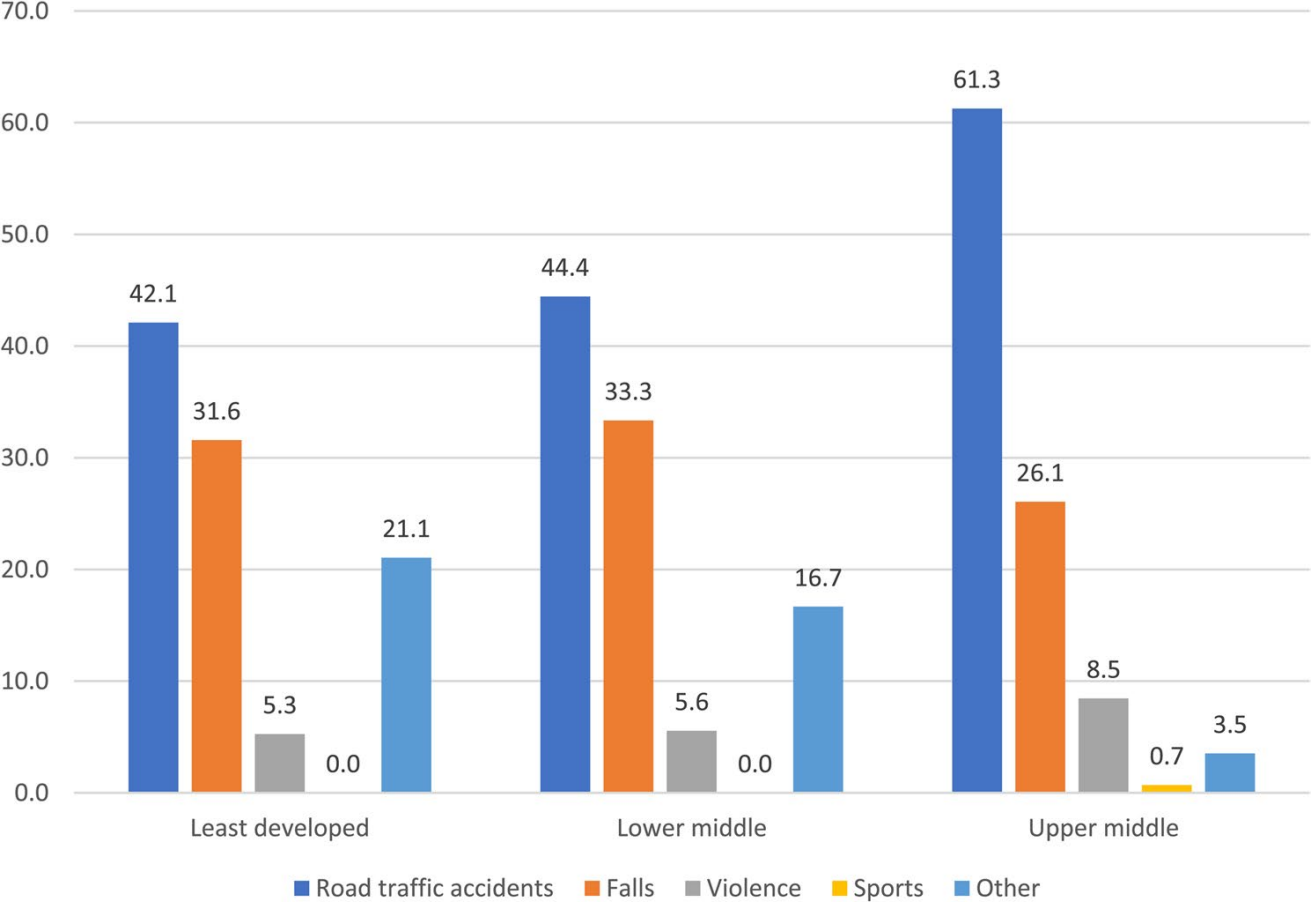
Causes of TBI as percentages by ODA regions

Healthcare professionals from least developed countries reported that the main cause of TBI (see Fig. 3) were road traffic accidents (42%) followed by falls (32%) and other (21%). A similar pattern was reported in lower middle income countries. Other causes reported included developmental, congenital conditions or cerebral malaria. Colleagues in upper middle income countries reported that the third most common cause of TBI was violence (9%) and were the only category to report sport (1%) as a cause of TBI.

### Funding for rehabilitation services in ODA regions

**Fig. 4:**
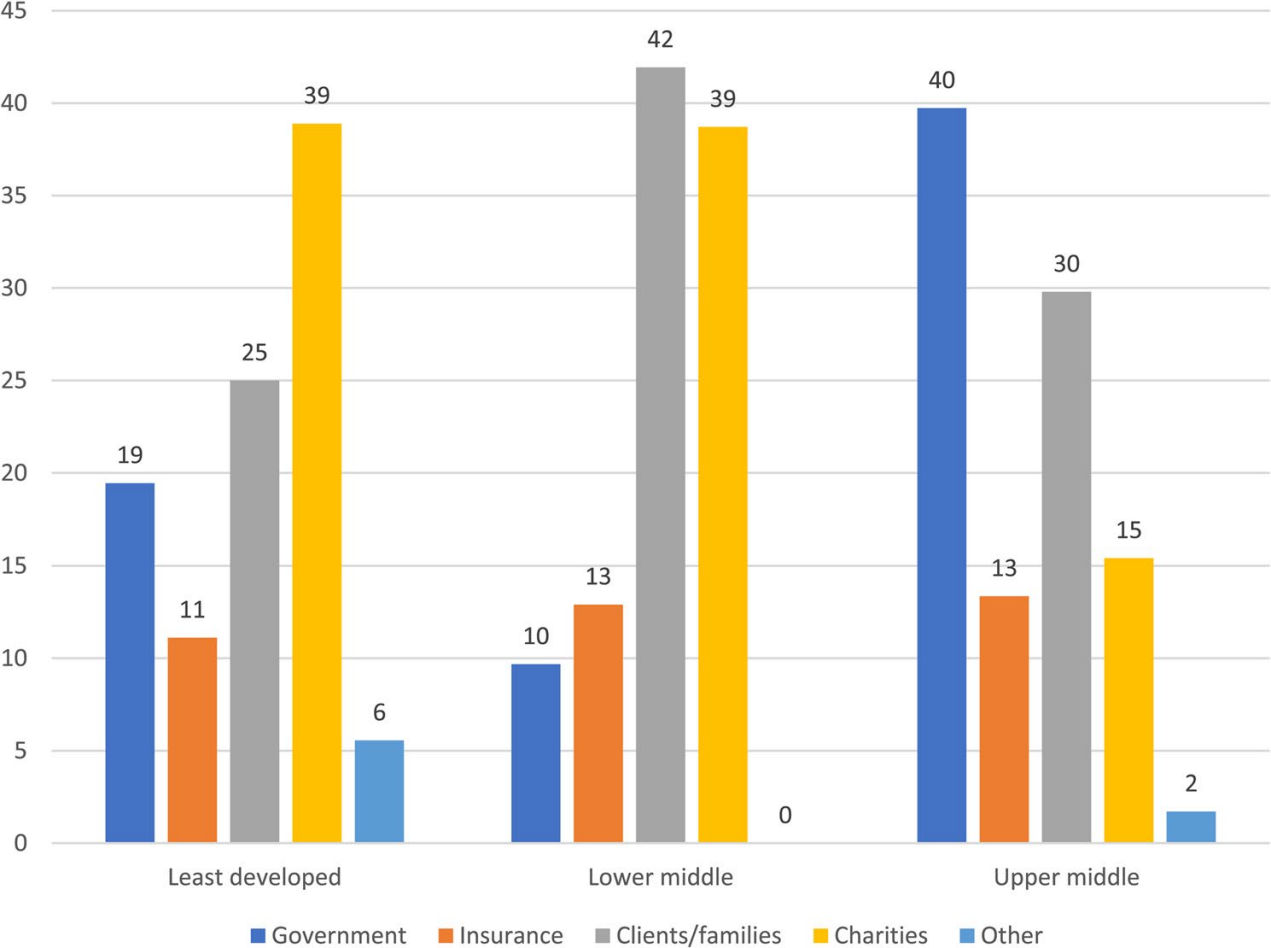
Funding rehabilitation services as percentages by ODA regions

When asked where funding for rehabilitation services was obtained (see Fig. 4), healthcare professionals from the least developed regions stated that the main sources were charities (39%), followed by clients/families (25%) and governments (19%). Those from lower middle countries reported that clients/families (42%) themselves paid for rehabilitation, followed by charities (39%) and insurance (13%). Colleagues from upper middle income regions reported that governments (40%) were the main source of rehabilitation funding followed by clients/families (30%) and charities (15%).

### Differences between ODA ranked countries on access to services

**Fig. 5:**
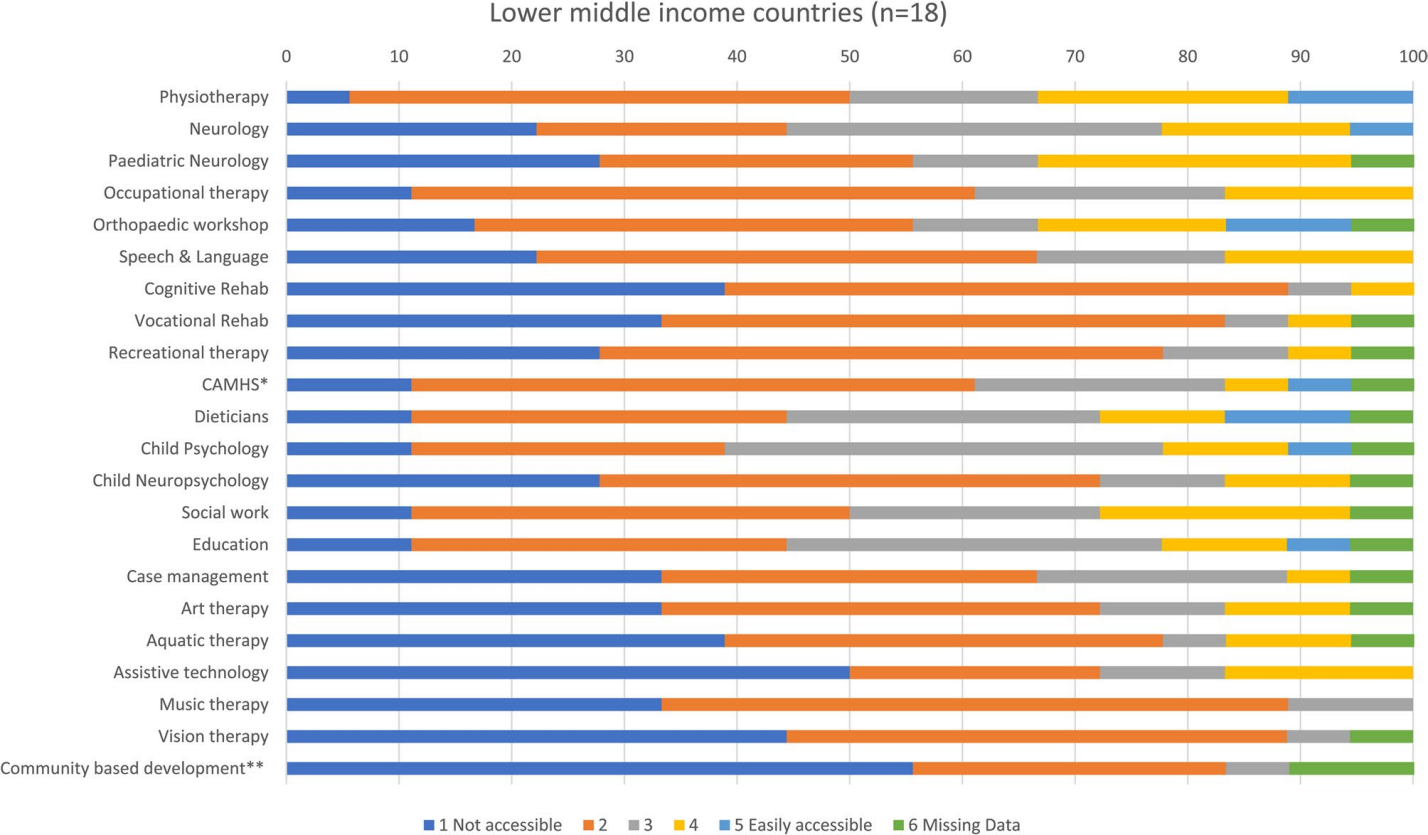
Stacked bar chart showing responses for access to services in lower middle income countries. *child and adolescent mental health services; **community based inclusive development programme

**Fig. 6:**
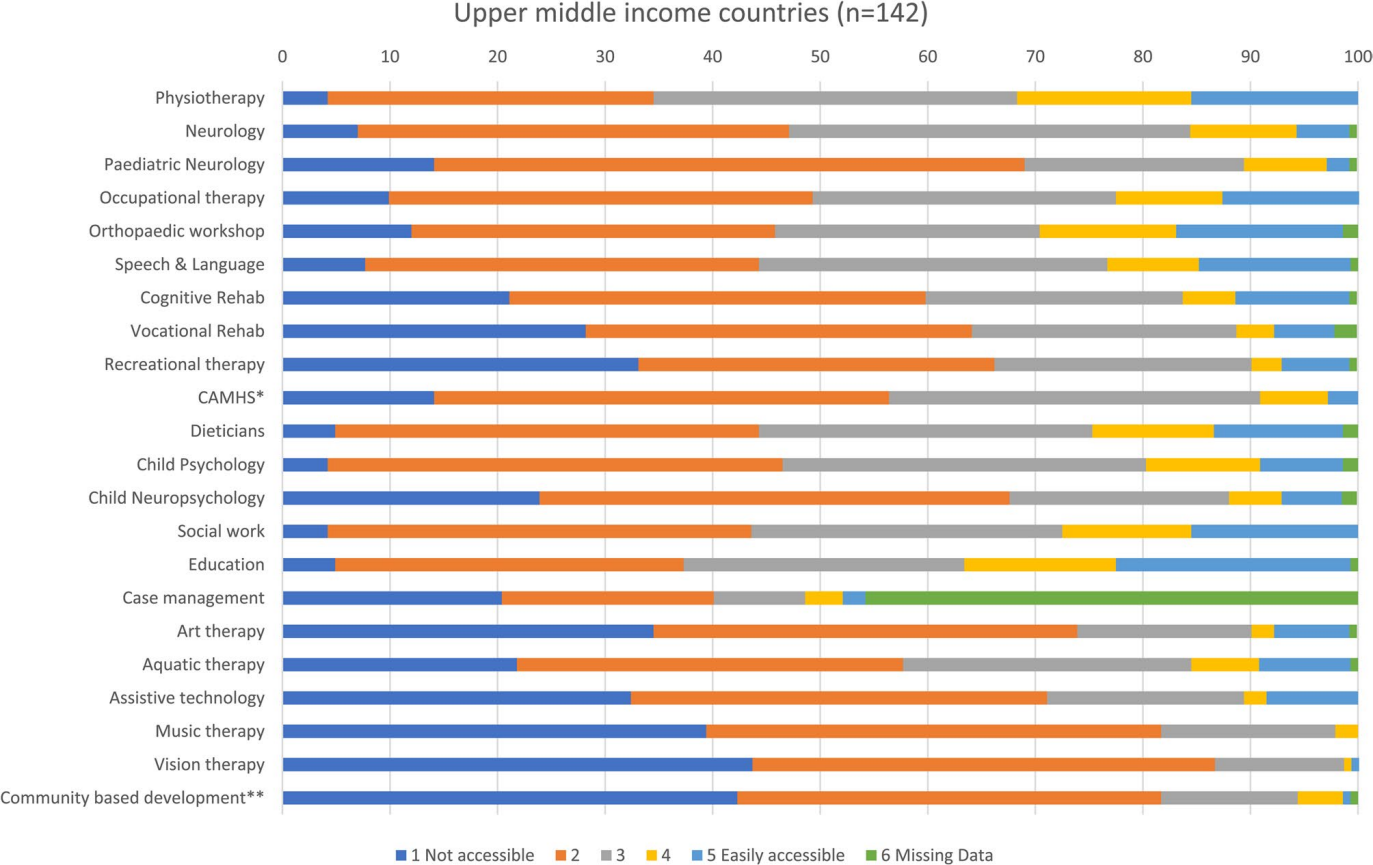
Stacked bar chart showing responses for access to services in upper middle income countries. *child and adolescent mental health services; **community based inclusive development programme

**Fig. 7:**
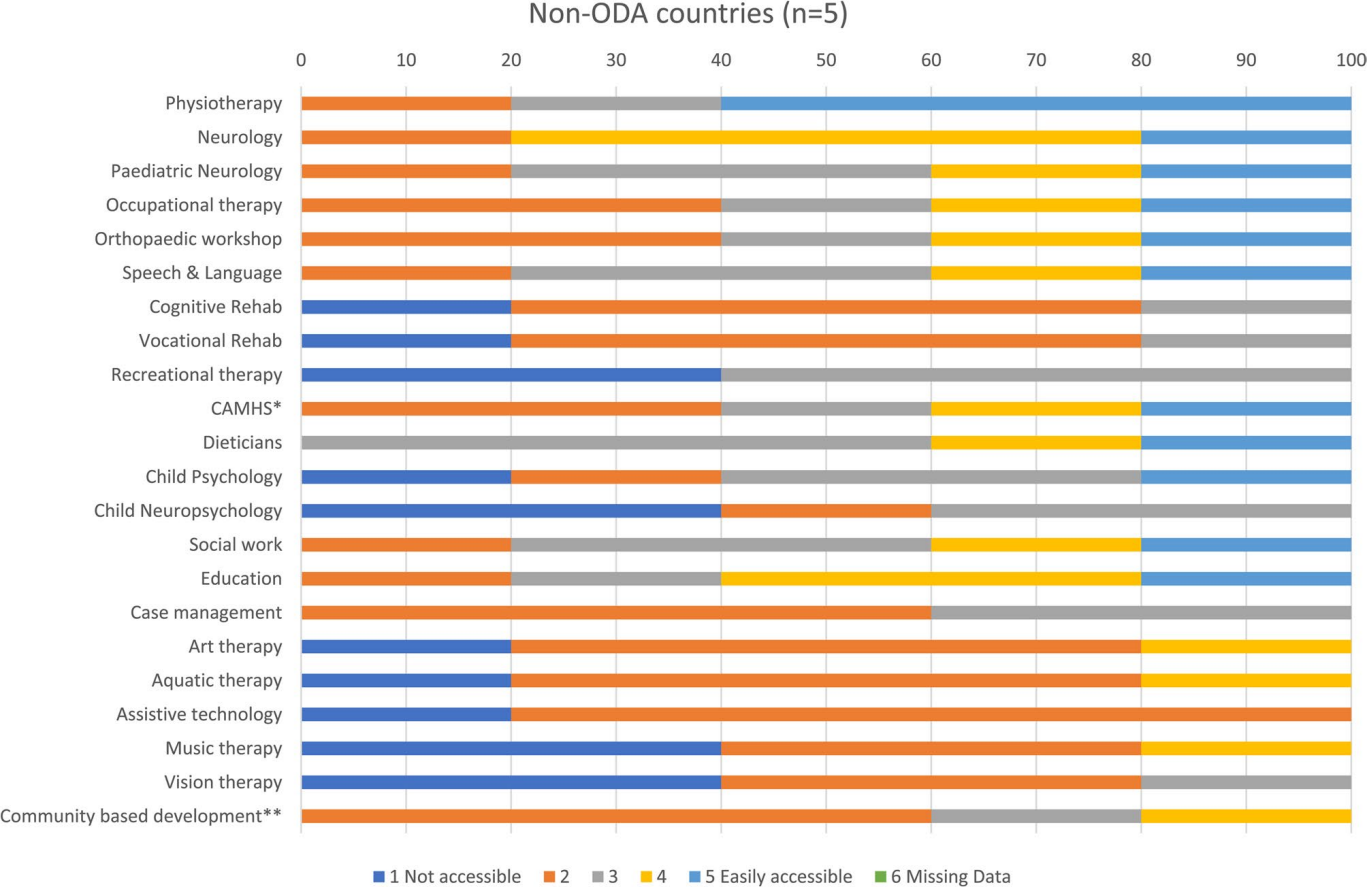
Stacked bar chart showing responses for access to services in non-ODA countries. *child and adolescent mental health services; **community based inclusive development programme

Figures 1, 5, 6, and 7 show access to services, expressed as percentages, in the least developed, lower middle, upper middle and non-ODA countries. Among least developed countries cognitive rehabilitation (69%), and aquatic therapy (68%), were the least accessible services. Healthcare professionals from lower middle income countries reported that it was most difficult to access community based inclusive development programmes (CBIDP) (56%), and assistive technology (50%) Participants from upper middle income countries reported having difficulty in accessing vision therapy (44%), and CBIDP (42%). Non-ODA countries were included here (Fig. 7) for the purposes of comparison and showed that these countries had greatest difficulty in accessing child neuropsychology (40%), recreational therapy (40%), music therapy (40%) and vision therapy (40%). As only 5 healthcare professionals were included in this category these findings should be treated with caution.

Independent samples Kruskal Wallis tests showed statistically significant differences in access for occupational therapy [H (2) = 10.73, *P* =.005], speech and language therapy [H (2) = 6.57, *P* = .038], cognitive rehabilitation [H (2) = 15.70, *P* < .001], vocational rehabilitation [H (2) = 9.69, *P* = .008], aquatic therapy [H (2) = 15.73, *P* < .001] and CBIDP [H (2) = 9.44, *P* = .009]. Physical therapy approached statistical significance [H (2) = 5.13, *P* = .077], as did art therapy [H (2) = 5.38, *P* = .068] and music therapy [H (2)= 5.27 *P* = .072].

Pairwise comparisons using Dunn’s test showed that these statistically significant differences lay between least developed and upper middle income countries (*P* = .001) for occupational therapy, lower middle and upper middle income countries (*P* = .050) for speech and language therapy, least developed and upper middle (*P* < .001), lower middle and upper middle income countries (*P* = .021) for cognitive rehabilitation, least developed and upper middle income countries (*P*= .003) for vocational rehabilitation, least developed and upper middle (*P* < .001), lower middle and upper middle income countries (*P* = .050) for aquatic therapy and least developed and lower middle (*P* = .003), least developed and upper middle income countries (*P* =.015) for CBIDP.

## Discussion

Rehabilitation following BI is crucial to ensure children and young people have positive outcomes [8, 13, 14]. In this study, physiotherapy was one of the most accessible rehabilitation approaches reported. This may be due to the emphasis in healthcare on patients becoming ambulatory as a first step in recovery [15]. However, the impact of BI on cognition has been consistently shown in research [16–18] and yet professionals from LMICs reported cognitive rehabilitation as one of the most difficult to access treatments. Other rehabilitation services which were not accessible, or were difficult to access, included vocational rehabilitation, art therapy, aquatic or hydrotherapy and music therapy. While some of these approaches may be considered non-essential, and so receive limited funding, they also require limited resources. Music and art therapy have been shown to be effective at improving cognitive outcomes (e.g. executive functioning) and are relatively low cost [19–21]. Therefore, these could be implemented relatively easily in under resourced areas, improving cognitive outcomes for children and young people with BI.

Participants from upper middle income countries reported that rehabilitation was funded via government, clients/families and charities. This pattern differed for least developed and middle income countries where charities and clients/families were the main sources of funding. A lack of sustained and centralised funding would impact on the ability of a rehabilitation service to deliver ongoing care and would reduce the likelihood of long-term provision. As an illustration, severe TBI is associated with significant economic burden (USD 76.5 billion) in both direct medical (USD 11.5 billion) and indirect (e.g. non-medical, lost productivity) (USD 64.8 billion) costs [22]. The cost of in-patient rehabilitation is thought to be around one third (USD 560) of the total daily cost (USD 1,562) of treatment for children with moderate to severe TBI [23]. The five-year cost of early rehabilitation following TBI has been estimated as USD 116,796 or USD 120,363 for delayed treatment per person [24].

Participants from LMICs reported hypoxia as the leading cause of BI. This is in contrast to what we see in developed countries where TBI is reported as the principle cause [25]. Rates of death due to labour-related intrapartum hypoxia are significantly greater in LMICs (4.8 per 1000 births) compared to higher income regions (e.g. UK 0.18 per 1000) [26, 27]. Hypoxic ischemic encephalopathy is a type of BI caused by a lack of oxygen to the baby’s brain at the time of birth [27]. Hypoxic injuries are thought to account for around 1 million such injuries and can be due to poor obstetric care [28]. In LMICs, hypoxia also occurs commonly in acutely ill children and those with respiratory infections, especially in children under 5 years of age [29, 30]. All participants reported road traffic accidents and falls as the greatest cause of TBI in children and young people. This is similar to what is reported in the literature in developed regions [31, 32]. Children who cycle may be struck by cars or be injured as passengers [32, 33]. Researchers have suggested that children should wear bicycle helmets to reduce the impact of being struck by cars [33]. To date, around 28 countries (out of 195) have instituted compulsory wearing of bicycle helmets in the face of evidence to their effectiveness at reducing injury [34]. Researchers have shown a 46% reduction in fatal crashes following the introduction of such legislation in Australia [35] and a reduction in head injuries [36]. Three LMIC (i.e. Namibia, Nigeria and South Africa) have introduced such laws [37].

The lack of recognition of sports as a cause of TBI in LMICs is concerning. It is widely recognised that sports can lead to concussion and TBI [38, 39]. Whilst the symptoms of concussion or mild TBI may resolve in the days or weeks following injury, in around 25–31% of such cases, symptoms are persistent and ongoing [40, 41]. Participants in this study may have focused more on the moderate to severe end of the injury spectrum and omitted milder injuries. Children and young people who are injured through road traffic accidents may receive more moderate or severe BI which would be clearly recognised by healthcare professionals due to their need for hospitalisation [42, 43]. Individuals who receive mild TBI, for which hospitalisation is often not required, may not be recognised and receive appropriate treatment. Participants in upper middle income countries reported that less than 1% of TBI were caused by sports suggesting that this is not a widely recognised vector for injury. As such, preventative or treatment options for milder injuries may not be considered.

### Strengths and limitations

As no previous study had sought to explore this topic in LMICs a new survey tool was developed. While this was pilot tested with healthcare professionals it was beyond the scope of this work to test its validity or reliability. However, pilot testing indicated a certain degree of face validity and participant responses showed that they understood its purpose. The survey was translated from English into three other languages to ensure optimal inclusion from Spanish, Portuguese and French speaking countries. It was not possible to recruit from French speaking countries, meaning that these are not represented in the current study. Further, it was not feasible to include participants who spoke other languages, due to resource constraints, which meant participation was limited to colleagues who understood English, Spanish and Portuguese. The online nature of the survey may have limited responses to professionals with internet access, which can be a problem in resource poor regions, or for those with limited service provision. Whilst equal representation was sought from all LMICs, certain countries were better represented than others (e.g. Brazil and Mexico). Gaining fewer responses from certain countries may skew perceptions of services and limit generalisability. In addition, there was some variety in the healthcare and other professional respondents. It is possible that the varying roles of these professionals impacted the extent to which they were aware of the accessibility of rehabilitation services in their country. Professionals were recruited from just under 23% of ODA recipient countries (32 out of 140). Whilst further participation would have been preferable it should be remembered that this is the only study to address this topic in LMIC.

### Recommendations

Relatively low-cost interventions such as music and art therapy could easily be incorporated into resource poor regions. Whilst music therapists are skilled practitioners, some benefits of music therapy can be achieved with relatively little training. For example, singing has been shown to improve speaking, voice quality [44] and mood [45] following BI. Voluntary sector organisations, carer support groups or trainee healthcare professionals could be upskilled to provide rudimentary music and art therapy.

There is urgent need for governments in LMICs to provide resources, for personnel, training and the development of services, to improve outcomes for children and young people with BI. Centralised and sustained funding is required to ensure that existing services are adequately supported and would allow for provision of access to those which are not currently offered or which are difficult to access. Governments should see this as an investment in the next generation of their population, given the positive outcomes these services can have on children and young people with BI. Not only would such investment improve outcomes for children and young people following BI, it would also reduce future dependence on healthcare and allow them to lead productive lives.

The long-term potential impact of sport related mild TBI on children’s lives should be highlighted. Whilst these children may not require hospitalisation they may experience persistent and detrimental symptoms which could reduce their life chances and quality of life.

## Conclusions

Many rehabilitation services for children and young people with BI in LMICs are difficult to access or are non-existent. Further resources are required from developed regions to support services in LMIC for these children. Improvements to obstetric care are needed to reduce hypoxic injuries at birth. Without such resource children and young people with BI will face poor outcomes and reduced life opportunities.

## Supplementary Information

Supplementary Material 1.

## Data Availability

Anonymised data for this study are available upon request to the corresponding author.

## Abbreviations

ABI: Acquired Brain Injury
BI: Brain injury
CBIDP: Community Based Inclusive Development Programmes
DAC: Development Assistance Committee
GNI: Gross National Income
LMIC: Low and Middle Income Country
ODA: Official Development Assistance
OECD: Organization for Economic Cooperation and Development
TBI: Traumatic Brain Injury
USD: United States Dollar
