# Supplementary material for: A multi-national cross-sectional exploration of rehabilitation services for children and young people following brain injury in low and middle income countries
Source: BMC Public Health. 2025 Nov 3;25:3739. doi: 10.1186/s12889-025-24933-0 (PMC12581435; doi:10.1186/s12889-025-24933-0)
Supplement: Supplementary file 1 — Supplementary Material 1. [file 12889_2025_24933_MOESM1_ESM.pdf]

Survey of rehabilitation services for children and young people following brain injury in Low and Middle Income Countries.

We would like to know what services you have available to you for supporting the rehabilitation needs of children and young people following brain injury. As such we are conducting a survey to establish the service needs in your country/region.

What is your profession? *Free text response*

What gender do you identify as? Man, Woman, Non-binary, Prefer not to say.

What is your highest qualification in your field? Doctorate, Postgraduate degree (e.g. MSc etc), Undergraduate degree (e.g. BSc etc), Diploma, Certificate

How many years have you been involved with rehabilitation? *0-5 years, 6-10 years, 11-15 years, 16-20 years, 21+ years*

If you have been involved with paediatric rehabilitation, how long have you worked in this field? *0-5 years, 6-10 years, 11-15 years, 16-20 years, 21+ years*

How many children and young with brain injury do you provide services every month (insert ranges) <20, 21-40, 41-60, 61-80, 80+

In which country are you working? *Free text response*

In which region of this country are you working? *Free text response*

In your region, who pays for rehabilitation provision? Government, Non-Government Organisations/Charities, Clients/families, Insurance, Other, Not applicable

Is the region where you work rural or urban? Rural, Urban

What is the main cause of brain injury in children and young people in your country

1. Traumatic brain injury
2. Brain infections
3. Cancer
4. Congenital conditions
5. Vascular conditions
6. Hypoxia
7. Other

What is the main cause of traumatic brain injury in children and young people where you work

1. Road traffic accidents
2. Falls
3. Violence
4. Sports
5. Other

In your country do you have specialised rehabilitation centres for children and young people with Brain injury? Yes, No

What are the main barriers for children and young people with brain injury to receive rehabilitation services in your country (can check more than one)

1. The cost of the services
2. Access to services
3. There are no professionals who provide such services
4. There are no rehabilitation centres in my country
5. Existing attitudes and beliefs about rehabilitation recovery
6. Lack of awareness of rehabilitation
7. Existing intervention programs are not adapted to where I live
8. Other (free text response)

Please rank, from 1-8 (with 1 being the most challenging and 8 being the least), what you consider the main barriers are for children and young people with brain injury to receive rehabilitation services in your country.

1. The cost of the services
2. Access to services
3. There are no professionals who provide such services
4. There are no rehabilitation centres in my country
5. Existing attitudes and beliefs about rehabilitation recovery
6. Lack of awareness of rehabilitation
7. Existing intervention programs are not adapted to where I live
8. Other (free text response)

Which of the following services for children and young people with brain injury do you have access to in your region?

Physiotherapy

1 2 3 4 5

Not accessible Easily accessible

Neurology

1 2 3 4 5

Not accessible Easily accessible

Paediatric neurology

1 2 3 4 5

Not accessible Easily accessible

Occupational therapy

1 2 3 4 5

Not accessible Easily accessible

Orthopaedic workshop

1 2 3 4 5

Not accessible Easily accessible

Physical therapy

1 2 3 4 5

Not accessible Easily accessible

Speech and language therapy

1 2 3 4 5

Not accessible Easily accessible

Cognitive rehabilitation

1 2 3 4 5

Not accessible Easily accessible

Vocational rehabilitation

1 2 3 4 5

Not accessible Easily accessible

Recreational therapy

1 2 3 4 5

Not accessible Easily accessible

Child and adolescent mental health

1 2 3 4 5

Not accessible Easily accessible

Dietitians

1 2 3 4 5

Not accessible Easily accessible

Clinical psychology

1 2 3 4 5

Not accessible Easily accessible

Clinical neuropsychology

1 2 3 4 5

Not accessible Easily accessible

Social work

1 2 3 4 5

Not accessible Easily accessible

Teachers/Schooling

1 2 3 4 5

Not accessible Easily accessible

Case management

1 2 3 4 5

Not accessible Easily accessible

Art therapy

1 2 3 4 5

Not accessible Easily accessible

Aquatic therapy

1 2 3 4 5

Not accessible Easily accessible

Assistive technology

1 2 3 4 5

Not accessible Easily accessible

Music therapy

1 2 3 4 5

Not accessible Easily accessible

Vision therapy

1 2 3 4 5

Not accessible Easily accessible

Community Based Inclusive Development Programme

1 2 3 4 5

Not accessible Easily accessible
